# Bipedality and hair loss in human evolution revisited: The impact of altitude and activity scheduling

**DOI:** 10.1016/j.jhevol.2016.02.006

**Published:** 2016-05

**Authors:** Tamás Dávid-Barrett, Robin I.M. Dunbar

**Affiliations:** a Department of Experimental Psychology, University of Oxford, South Parks Road, Oxford OX1 3UD, UK; b Institut für Weltwirtschaft, Kiellinie 66, D-24105 Kiel, Germany; c Department of Computer Sciences, Aalto University, Espoo, Finland

**Keywords:** Australopiths, Thermoregulation, Incident radiation, Ambient temperature, Activity patterns

## Abstract

Bipedality evolved early in hominin evolution, and at some point was associated with hair loss over most of the body. One classic explanation (Wheeler 1984: J. Hum. Evol. 13, 91–98) was that these traits evolved to reduce heat overload when australopiths were foraging in more open tropical habitats where they were exposed to the direct effects of sunlight at midday. A recent critique of this model (Ruxton & Wilkinson 2011a: Proc. Natl. Acad. Sci. USA 108, 20965-20969) argued that it ignored the endogenous costs of heat generated by locomotion, and concluded that only hair loss provided a significant reduction in heat load. We add two crucial corrections to this model (the altitude at which australopiths actually lived and activity scheduling) and show that when these are included there are substantial reductions in heat load for bipedal locomotion even for furred animals. In addition, we add one further consideration to the model: we extend the analysis across the full 24 h day, and show that fur loss could not have evolved until much later because of the thermoregulatory costs this would have incurred at the altitudes where australopiths actually lived. Fur loss is most likely associated with the exploitation of open habitats at much lower altitudes at a much later date by the genus *Homo*.

## 1 Introduction

Brain tissue is extremely sensitive to heat, and must be kept within very narrow tolerances to avoid rapid cell death (Precht and Brück, 1973). Mammals have evolved a number of strategies to reduce heat accumulation in the brain when they occupy open habitats subject to high levels of direct radiant heat from the sun, especially at midday in the tropics when the sun is directly overhead. Such strategies include the evolution of a complex nasal rete that allows heat exchange between arterial and venous blood (many antelopes [Maloiy et al., 1988]), long muzzles that allow cerebral blood to be cooled by panting (baboons: Hiley, 1976), dense coats that keep incident radiation from the sun away from the skin surface (klipspringer antelope: Dunbar, 1979) and behavioural strategies such as seeking dense shade during the heat of the day (reedbuck: Roberts and Dunbar, 1991; baboons: Hill, 2006).

In a seminal series of papers, Wheeler, 1984, Wheeler, 1990, Wheeler, 1991a, Wheeler, 1991b, Wheeler, 1992 used a physiological model to argue that bipedal locomotion and hair loss in early hominins might have been an adaptation to reduce incident heat load when foraging in more open habitats. Unlike their great ape sister species that remained within the tropical forests, early hominins began to make increasing use of forest edge and more open woodland (but probably not open savannah) habitats where exposure to the direct rays of the sun was considerably greater, especially during the hottest times of the day when the sun is overhead. The australopiths and their allies were uncontroversially bipedal by at least 5 Ma (millions of years ago) (Pickford et al., 2002, Lovejoy et al., 2009) and more controversially so as early as 6 Ma (Brunet et al., 2002), although it is generally accepted that the earliest taxa were not particularly efficient bipeds (and may have used bipedalism for rather different purposes in a more semi-arboreal lifestyle: Thorpe et al., 2007, Crompton et al., 2008).

Recently, Ruxton and Wilkinson, 2011a, Ruxton and Wilkinson, 2011b, introduced an amendment to the Wheeler model by noting that, in addition to the exogenous heat load from the sun, an active hominin would generate much endogenous heat from walking. Including a factor for endogenous heat generation adds substantially to the heat load of bipedal hominins, effectively removing the advantage that Wheeler, 1984, Wheeler, 1990, Wheeler, 1991a, Wheeler, 1991b, Wheeler, 1992 claimed for it. Ruxton and Wilkinson, 2011a, Ruxton and Wilkinson, 2011b argue that only hair loss could have been selected for in terms of heat load reduction. They conclude that, with little or no thermal advantage, bipedalism must have evolved for some other reason.

**Figure 1:**
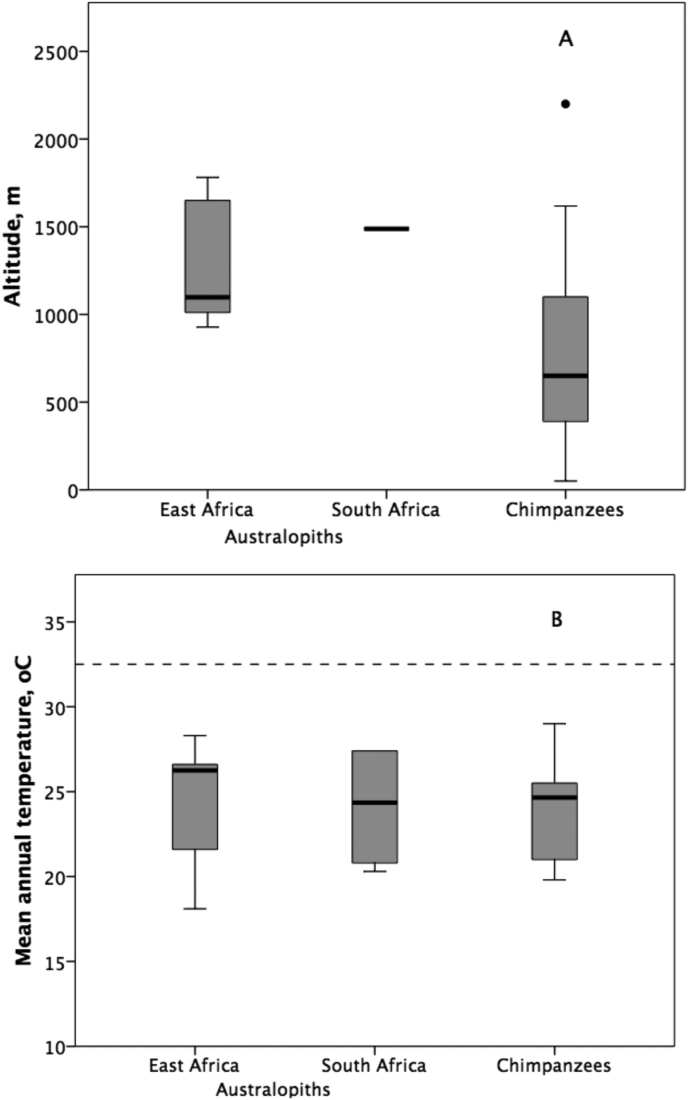
(A) Median (±50% and 95% ranges) altitudes of East African (*n* = 9) and South African (*n* = 3) fossil australopith (*Australopithecus* and *Paranthropus*) sites, compared to modern chimpanzee study sites (*n* = 14). Outliers are indicated by a solid dot. Altitudes for fossil sites are corrected from current altitude to reflect the fact that the Rift Valley floor is now much lower than it was c. 3 Ma (see text for details; fossil sites are listed in SOM Table S1). Sources: Lehmann et al., 2007, Bettridge and Dunbar, 2012. (B) Median (±50% and 95% ranges) mean annual temperature at fossil australopith sites in East Africa (*n* = 35 horizons) and South Africa (*n* = 5) and for modern chimpanzee populations (*n* = 14), compared to the mean temperature assumed in the Ruxton and Wilkinson, 2011a, Ruxton and Wilkinson, 2011b model (dashed line). The value for the Ruxton–Wilkinson model is the mean value integrated across their diurnal temperature distribution; values for fossil sites are for individual horizons, with temperatures being estimated from modern sites matched as closely as possible for faunal profile (see SOM Table S1). Sources: australopith sites (Bettridge and Dunbar, 2012); chimpanzees (Lehmann et al., 2007).

The Ruxton and Wilkinson, 2011a, Ruxton and Wilkinson, 2011b (RW) model made a number of implicit assumptions that have radical implications for heat load. One assumption was that the temperature regimes under which australopiths lived were, in effect, those at sea level at the equator (where maximum day time temperatures can rise to 40 °C or above). The evidence from the fossil record suggests that all currently known australopith sites lie at altitudes above ∼1000 m above sea level (asl) (Fig. 1A; Supplementary Online Material [SOM] Table S1) and so models of heat load must take this into account. Indeed, even chimpanzees do not live at sea level today, but typically live at altitudes between 500 and 1000 m asl. The Rift Valley floor has dropped some considerable distance since 3 Ma as a result of tectonic activity (Partridge, 1997), and current australopith sites in Ethiopia are estimated to be as much as 1000 m below their original position (Bonnefille et al., 1987), while those in northern and central Kenya are about 500 m lower (Dowsett et al., 1999). Only the South African sites are at approximately the same altitude as they were (they are thought to have dropped by only about 90 m). All the sites in Figure 1A have been corrected by these amounts. The mean altitude (corrected for tectonic effects) for South African sites is 1488 m asl, for East African sites 1313 m, and for the chimpanzee sites 819 m. Figure 1A suggests that modern day chimpanzees live at lower altitudes than australopiths did. However, this is somewhat misleading if taken at face value: current temperatures are 2–3 °C lower than they were in the Pliocene (Demenocal, 1995, Bartoli et al., 2005, Robinson et al., 2008). Crucially, the mean Plio-Pleistocene temperatures for the East African australopith sites (from Bettridge and Dunbar, 2012; for details, see SOM Table S1) do not differ from those for modern day chimpanzee sites (from Lehmann et al., 2007) (Fig. 1B: F_1,48_ = 0.53, p = 0.470).

Palaeoanthropologists often advise caution when drawing biogeographical conclusions on the basis of fossil evidence: absence of evidence, we are reminded, is not necessarily evidence for absence. However, in this case, there are three reasons for thinking that the claim that australopiths did not live below ∼1000 m asl is robust.

First, there are low altitude sites from this period that have not yielded hominins despite the fact that they contain monkeys, and are thus evidently suitable for primates. The Chiwondo beds of Lake Malawi (∼500 m asl), for example, contain mainly papionins, whereas the high altitude specialist *Theropithecus* (Dunbar, 1998) is rare (Frost and Kullmer, 2008) despite being common at most of the contemporary classic higher altitude australopith sites in both eastern and southern Africa. Hominins (*Paranthropus bosei* and *Homo rudolfensis*) only appear late in the sequence after 2.5 Ma (Sandrock et al., 2007, Frost and Kullmer, 2008, Bocherens et al., 2011), coincident with the dramatic decline in temperature associated with the 2.5 Ma climatic event. Other low altitude sites that contain monkeys but no australopiths include Ahl al Oughlam (100 m asl) in Morocco, dated to ∼2.5 Ma (Alemseged and Geraads, 1998), the late Miocene Libyan site of Qasr as Sahabi (0 asl; Boaz et al., 2008) and the late Miocene to early Pliocene site of Langebaanweg in the Western Cape (30 m asl; Hendey, 1981). Additionally, while there is uncontroversial evidence for coastal migrations of ungulates (but not australopiths) between Eurasia and sub-Saharan Africa during this period (e.g., Bibi, 2007). In contrast, the appearance of *Homo* around 2.8 Ma is marked in due course by the presence of *Homo* fossils at various sites around the Mediterranean (Ternifine: Geraads et al., 2008; Kocabas, Turkey: Kappelman et al., 2008), as well as evidence of Olduwan/pre-Acheulian industries (e.g. Ain Hanech at∼650 m asl in Algeria, and various litoral sites in the Magreb and coastal Morocco and Algeria, including the aforementioned Ahl al Oughlam: Biberson, 1961, Sahnouni, 2006). In addition, there is the indirect evidence for coastal occupation implied by the early *Homo ergaster*/*erectus* migrations out of Africa into Eurasia – but still no low altitude australopith fossils even though these continued to be around for some considerable time.

Second, models of time budgets for australopiths suggest, using an entirely different approach, that they could not survive in high temperature, low altitude habitats (Bettridge, 2010), whereas fossil papionins were able to occupy a much wider range of habitats (Bettridge and Dunbar, 2012). Third, estimates of the mean annual temperature for fossil sites (estimated by faunal profile matching to modern sites [Bettridge and Dunbar, 2012]) indicate that the temperature range within which australopiths are known to have lived is identical to that for modern chimpanzees: the mean temperature is ∼25 °C for all three taxonomic groups (Fig. 1B). Given the climatic cooling that has occurred since 3 Ma, it follows that australopiths would have occupied sites that are around 500–600 m higher in altitude than those currently occupied by chimpanzees (note that even these do not occupy coastal habitats). This suggests that the mean temperature value of 32.5 °C assumed in the RW model (the dashed line in Fig. 1B) significantly exceeds the range of values at which both chimpanzees and australopiths live.

The second assumption made in the Ruxton–Wilkinson model is that australopiths were continuously active throughout the day, and would thus have generated substantial additional endogenous heat from walking. While accounting for the extra endogenous heat load from walking is important, the assumption that animals are continuously walking is incorrect. Most primate species spend only about 20% of their day walking (including both travel and foraging movements within food sites) (Dunbar et al., 2009). The mean actual travel time for chimpanzee populations, for example, is 17.1% (range 7.5–27.6%: Lehmann et al., 2007), while baboons – the taxon with the longest day journeys that make most use of wooded and grassland-edge habitats – devote only 20–35% of their day to moving (Bettridge et al., 2010). Estimates from time budget models for the australopiths suggest that they would have devoted only about 16% of their day to travel (Bettridge, 2010). More importantly, living primate species, such as baboons, that occupy similar forest edge and riverine habitats to those occupied by australopiths typically travel to and from foraging areas only in the morning and late afternoon/evening (Hill, 2006), precisely the times when the RW model suggests that australopiths might have had sufficient spare thermal capacity to allow travel. For most tropical species, the hottest part of the day is spent resting in shade to minimize endogenous heat production (Roberts and Dunbar, 1991, Hill, 2006). Indeed, animals typically rest rather than groom when temperatures rise significantly above their thermoneutral zone (Hill, 2006).

A third implicit assumption made by both the Wheeler and the RW models is that animals do not incur any thermal costs overnight, and the night time thermal environment is therefore ignored. In fact, even at altitudes of ∼1000 m asl in East Africa, ambient night time temperatures are commonly as low as 10 °C, and can fall to as low as 5 °C on occasion (R. Dunbar, unpublished field data), imposing substantial thermal costs on hairless animals. This is not usually an issue at sea level, where ambient night time temperatures remain high and heat-shedding is more often a problem than heat loss. But at high altitudes, it is a problem, and if animals can lose heat overnight a heat load model needs to cover the full 24 h diurnal cycle.

In this paper, we explore the consequences for the RW model of accounting of adjusting altitude, travel time and night-time thermal costs. We show that when the model is corrected for altitude and activity scheduling, both bipedalism and hair loss would have generated consequential heat load savings, just as Wheeler, 1984, Wheeler, 1990, Wheeler, 1991a, Wheeler, 1991b, Wheeler, 1992 argued. However, cool night time temperatures would have made it impossible for substantial hair loss to have evolved in species occupying the sites where australopiths appear to have lived in the absence of cultural (e.g. shelter, clothing) or other behavioural (nesting, group sleeping) developments.

## 2 The model

Our basic model is identical to the RW (2011a) model, which itself was a variation of the Wheeler model (Wheeler, 1984, Wheeler, 1990, Wheeler, 1991a, Wheeler, 1991b, Wheeler, 1992). We give the full set of equations for the model in the Appendix. Here, we give only a brief summary of the main details of the parameterization.

In the revised model we present here, the total heat load (*Q*_*total*_) that the animal's body has to deal with is a combination of two main factors, environmental heat load and internal metabolic heat load. The environmental heat load has six main components:

(i) effect of outside air temperature, dependent on the stature of the animal and the time of day;
(ii) effect of air moving past the body (in the Wheeler model, this is due to wind; in the RW version it is due to the body moving);
(iii) effect of short and long wave radiation, dependent on the time of day;
(iv) effect of the pelt, and the degree to which it absorbs (short wave) radiation;
(v) effect of the total body surface, at the same time both absorbing solar radiation and allowing the body to cool by both convention and evaporation; and
(vi) effect of the proportion of the body that is exposed to direct sunlight.

Internal metabolic heat load has four key components:

(i) effect of basal metabolic heat production;
(ii) effect of metabolic heat generated by the body while active (due to movement);
(iii) effect of heat loss through respiration; and
(iv) effect of heat loss through sweating.

To adjust for the additional refinements, we:

(i) reduced the total proportion of the day spent moving to 16%, and allocated that evenly across the hours 06:00–11:00 and 14:30–18:00 (with travel time = 0% across the intervening midday period);
(ii) recalibrated the diurnal temperature regime so that it has a maximum midday value of 33.5 °C instead of 40 °C (where 33.5 °C is the maximum that would be given by the diurnal temperature regime in the RW model, but set to yield a mean temperature across the day of ∼26 °C, the value for the upper 75th centile in Fig. 1B; see SOM Table S1).

In addition, to allow for the fact that most tropical animals, including baboons that occupy a habitat similar to that argued for australopiths, rest in shade during the hottest part of the day (Hill, 2006), we also separately adjusted the model such that:

(iii) animals incur limited incident radiation (that is, they are in at least partial shade) over the midday period (11:00–14:30) when ambient temperatures are at their highest.

### 2.1 Parameterisation

In line with the Wheeler and the RW models, we assume that the female and male animals are different in leg length and body mass (52 cm and 30 kg, versus 72 cm and 55 kg respectively). Apart from these and the physical constraints of solar radiation, the original RW equation system, Equations (1), (2), (5), (6), (7), (8), (9), (10), (11), (12), (13), (14), (15), (16), (17), (18), (19) in the Appendix, assume the following:

The percentage of the day spent moving is 100%;

The temperature at 2 m, $\tau_{200}$, is assumed to be 40 °C;

The portion of the day the hominins spend in shade is assumed to be 0%.

We also included wind speed (based on sampled wind speeds from an East African Rift Valley site, Gilgil, at ∼622 m asl: mean wind speed across the day = 0.46 m/s, *n* = 2910 records [R. Dunbar, field site records]) in the model, but it made little difference to the results, so we disregarded it in the final version. Wind speed is therefore assumed to be 0 m/s.

We first ran the model both in the original RW formulation (to confirm that we obtained the same results as Ruxton and Wilkinson) and with the new adjustments we outline below.

#### 2.1.1 Refinement 1

To correct for the fact that hominins were more likely to spend 16%, rather than 100%, of the day moving (Bettridge, 2010), and confined this to the morning and afternoon periods with a 3.5 h rest period (as predicted by the australopith time budget model [Bettridge, 2010]) over midday, we allocate the overall daily average of 16% travel to the morning and afternoon periods. For computational simplicity, we assume this travel time is evenly distributed within these 12–3.5 = 8.5 morning and afternoon hours, which means that (16% × 12)/8.5 = 22.6% of each hour would be spent moving, while the animals are assumed to be effectively motionless over the 3.5-h midday period. Since, in the RW equation system, speed of travel and percentage of time spent travelling transform linearly, we opted to simplify the calculation by simply adjusting the speed of travel variable, *v*, as follows:

$$\text{For}\;0600-1100\;\text{h}\;\text{and}\;1430-1800\;\text{h:}\;\text{v}=.226\text{*}1\text{.}7\sqrt{\text{L}}=0.384\sqrt{\text{L}}$$

For1100−1430h:v=0

(NB. In the calculations we assume that the posture is maintained during the resting period, i.e., quadrupedals stay quadrupedal, bipedals stay bipedal. This is for simplicity, and does not affect the model's results.)

#### 2.1.2 Refinement 2

From Figure 1A, we take an altitude of 1000 m asl as the lowest altitude at which australopiths lived, and we use the standard environmental lapse rate of 6.49 °C temperature drop per 1000 m (Jacobson, 2005) to calculate the baseline temperature for the model. This is slightly more conservative than the 7.5 °C difference that would be implied by the mean values in Figure 1B. Moreover, the value of 6.5 °C is derived from climatological theory and is thus not open to the methodological criticisms that fossil habitat climate estimates might be. Hence, in this refinement, we use the base temperature parameter of $\tau_{200}=33.51{}^{\circ}$. In effect, we consider a worst case scenario for the australopiths: any altitudes above 1000 m represent increasingly benign thermal conditions. (NB: solar irradiance also increases with altitude and will add to the thermal load. To check for this, we ran some additional simulations that included this effect [data not shown], but, at the altitudes occupied by australopiths, the effect is too small to alter the qualitative results of the model, and thus we do not include it in the model.)

#### 2.1.3 Refinement 3

To account for hominins sheltering in shade during the hottest part of the day, we assumed that the short wave radiation is reduced by 50% in this time period. For computational convenience, we have assumed that this midday break is centred on 13:00 h (when the RW temperature curve peaks), and runs from 11:00 to 14:30 h. In practice, tropical temperature regimes are not strictly symmetrical in the way Ruxton and Wilkinson (2011a) assumed, but are temporally displaced so that the hottest part of the day is usually 12:00–16:00 h. However, for computational convenience, the RW model assumes that the temperature regime is perfectly symmetrical, and we will follow suit since this window covers the period when temperature is maximized in the model. Hence,

$$S=\{\begin{array}{c} 865\;\text{Sin}\left(\frac{\pi \left(t-6\right)}{12}\right)\text{if}\;t<11\;\text{or}\;14\text{.}5<t\\ 0.5\cdot 865\;\text{Sin}\left(\frac{\pi \left(t-6\right)}{12}\right)\text{if}\;11<t<14\text{.}5 \end{array}\text{.}$$

#### 2.1.4 Refinement 4

Finally, we extended the model to cover the full 24-h day/night cycle. Rather than making arbitrary assumptions about the diurnal pattern of temperature change, we use diurnal temperature profiles for three contemporary East African sites (Lodwar, 507 m; Kisumi, 1171 m; and Nakuru, 1850 m) that bridge the minimum altitude occupied by australopiths. For each site, we averaged hourly temperature across the past 25 years (NOAA, 2014) and used these values to parameterize the model. We assumed that there is zero sunshine and zero movement before 06.00 h in the morning, and after 18.00 h in the afternoon; we also assumed that during the day the animals move on average 16% of the time, concentrated in the morning and evening.

## 3 Results

**Figure 2:**
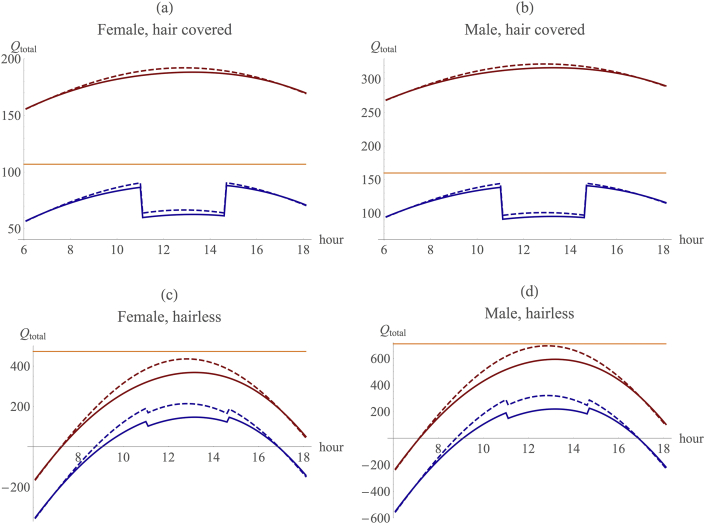
The combined effect of the re-parameterisation of the Ruxton–Wilkinson (RW) model: walking is limited to 16% of the day and confined to the morning and evening, and the altitude is at 1000 m asl. Separate values for the impact of the individual parameters are given in Fig. 3. X-axis: time of day, y-axis: total heat load (*Q*_*total*_). Red: RW model baseline; blue: RW model with all three additional refinements included. Dash: quadrupedal; continuous: bipedal. Orange: heat dissipation limit (from RW model). (For interpretation of the references to colour in this figure legend, the reader is referred to the web version of this article.)

Our version of the model (see also SOM Tables S2–S4 and Figs. S1–S5 for additional model results and sensitivity analyses) replicated exactly the results obtained by Ruxton and Wilkinson (2011a) during the same standard 12 h day that they used (Fig. 2, red lines). Thus, we can be certain that any differences that might emerge between their results and ours cannot be due to differences in the way the two models are built. We then consider the impact of each of the additional refinements on heat load one by one. In each case, we consider the two sexes separately, and examine the heat load for a quadruped versus a biped, and a haired (100% hair cover) versus a partially haired (15% hair cover) individual, just as Ruxton and Wilkinson (2011a) did.

**Figure 3:**
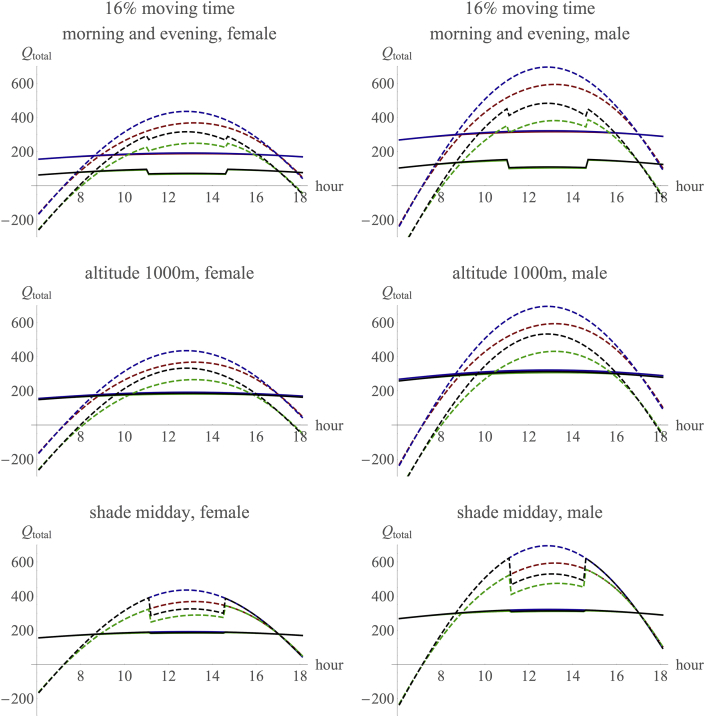
Comparing the three refinements added to the RW model individually shows that each reduces total heat load. Left column: females; right column: males. Rows: travelling only 16% of the day concentrated in the morning and evening, altitude at 1000 m, and hiding in shade at midday (11.30–14.30 h) when ambient temperatures are at their highest. Midday is defined as 11:30–14:30 h. Continuous line: 100% hair cover; dashed line: 15% hair covered; blue: RW quadruped; red: RW biped; black: modified model quadruped; green: modified model biped. (For interpretation of the references to colour in this figure legend, the reader is referred to the web version of this article.)

In each case, correcting the parameterisation of the original model separately for the lower travel time and higher altitude results in a considerable reduction in heat load compared to the RW model (Fig. 3, SOM Table S2). With or without the midday rest effect, the combined effect of the travel time and altitude refinements yields a marked shift in the results from those predicted by the RW model (Fig. 2). (Note the scale difference in the upper [furred] and lower [hairless] panels in Fig. 2: the difference between the revised model and the original RW values is in fact of the same magnitude in the two conditions.) Adjusting for the use of shade at midday would add a further modest reduction in heat load during the hottest part of the day (Fig. 3).

An animal's ability to survive in open/woodland habitats depends ultimately on how much of this additional heat can be shed across the day (Ruxton and Wilkinson, 2011a). We use the RW model assumption that female and male hair-covered hominins could shed 107W and 160W, respectively, by heat exchange with the environment, while hairless females and males could shed 473W and 710W, respectively, mainly due to the additional benefits of sweating (Ruxton and Wilkinson, 2011a, Ruxton and Wilkinson, 2011b, Wheeler, 1984, Wheeler, 1990, Wheeler, 1991a, Wheeler, 1991b, Wheeler, 1992). Note that the benefits of sweating only accrue to hairless animals, since, for furred animals, sweating simply cools the tips of the fur and not the skin beneath and is thus to no purpose; only if the fur is completely soaked is there any consequential benefit from sweating (Mount, 1979, Gebremedhin and Wu, 2001). These heat dissipation limits are represented by the orange lines in Figure 2: it is assumed that the animal is unable to shed heat above this limit, and as a consequence it overheats and dies.

Four points may be noted about the results presented in Figure 2, Figure 3. First, the adjusted model indicates that both haired and hairless animals would be able to lose sufficient heat by heat-dumping mechanisms like evaporative cooling and convection to maintain thermoneutrality throughout the day. However, in the hairless case, this comes at a substantial cost of water loss. Second, despite this, there might be an advantage to being haired because furred animals have an absolutely lower heat load throughout much of the sunny part of the day, and especially at midday when temperatures are at their highest.

**Figure 4:**
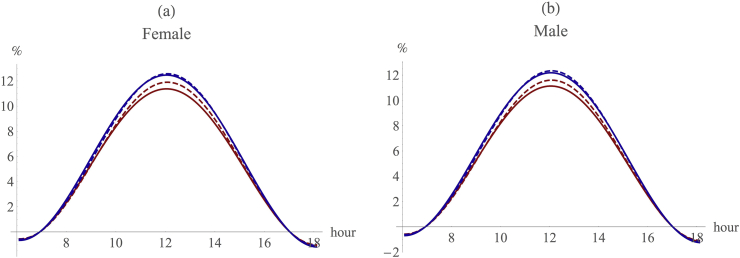
The relative advantage of switching from a quadrupedal to a bipedal stance for (a) females and (b) males, respectively. X-axis: time of day; y-axis: relative fall in heat load due to switching to bipedality. Red: RW model baseline; blue: RW model with all three additional refinements included. Dash: hairless; continuous: hair covered. (For interpretation of the references to colour in this figure legend, the reader is referred to the web version of this article.)

Third, there is always an advantage in being bipedal, especially over the midday period, and this is actually true of both the original RW model and our adjusted model. Notice that the advantage from being bipedal is substantially bigger in absolute terms for hairless animals than it is for furred ones, although the proportional gain over baseline is similar in both cases. This advantage is especially large at midday when heat stress is at its maximum: the thermal advantage from bipedality at midday is more than 11% in the RW baseline model, and more than 12% when taking into account the additional refinements we propose (Fig. 4). In other words, there is always a benefit to be gained from being bipedal rather than quadrupedal in these environments (Fig. 4, SOM Table S3). From an evolutionary ecological perspective, any such savings translate directly into fitness gains because the animal is under less stress and has to use less energy in heat dissipation (which becomes increasingly energetically costly as the thermal environment exceeds the animal's thermoneutral zone [see Mount, 1979]).

Fourth, while in absolute terms there is a benefit from being hairless over being hair covered (the difference between heat load and the capacity to shed heat is absolutely greater for hairless animals than for hair covered ones, as shown in Fig. 2), hairless animals incur a substantial cost in terms of heat loss early and late in the day that furred animals do not, and this would act as an important drag on the advantages of evolving hairlessness. Since nights are much cooler than the day at altitudes of 1000 m asl and above (at this altitude, the day–night temperature differential can be as much as 15 °C at the equator [R. Dunbar, unpublished field data]), the night-time cost of losing hair increases further at this altitude (SOM Table S4).

**Figure 5:**
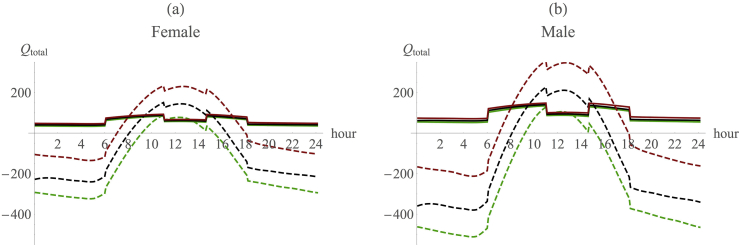
Heat load over the 24 h day for (a) females and (b) males in the quadrupedal case using current day temperature data from three locations: Lodwar (red) at 507 m, Kisumi (black) at 1171 m, and Nakuru (green) at 1850 m. X-axis: time of day; y-axis: total heat load (*Q*_*total*_). Continuous line: hair covered; dashed line: hairless individuals. (For interpretation of the references to colour in this figure legend, the reader is referred to the web version of this article).

To explore this issue in more detail, we estimated net heat load across the whole 24-h night/day cycle for haired and hairless quadrupedal individuals using the thermal profiles for the three contemporary East African sites (Lodwar, Kisumi and Nakuru). The results (Fig. 5) show that hair covered individuals remained in positive heat balance even at night when ambient temperatures fall and there is no sunshine and no additional body heat generated from movement. In contrast, individuals that have only 15% hair cover run into considerable heat deficit during the night, and the deficit increases with altitude: even at an altitude of 1000 m, females would require an additional 3500 kcal a day, while males would need 5600 kcal, to offset the costs of night time heat loss (SOM Table S4). Set against a daily energy requirement for australopiths of 1250 kcal for females and 1740 kcals for males (Aiello and Wells, 2002) and a time budget with no spare capacity at all for extra feeding (Bettridge, 2010), australopiths would have been incapable of balancing their energy budgets if they had been hairless at the altitudes where they seem to have.

Finally, as a check on the travel time assumption, we used the model to calculate the maximum time that hominins could spend travelling in each hour of the day without exceeding their heat loss capacity. The results suggest that, in the limit, they could in fact devote up to 40% of the time to travel during the middle hours of the day and up to 60% to travelling during the morning and evening hours without compromising their thermal balance (SOM Fig. S3). No primate species spends as much as 40% of the time travelling, not least because the demands of other time budget components (in particular, feeding and resting) severely restrict the time available for travel (Dunbar et al., 2009). The bottom line, however, is that the australopiths would have been well within their thermal limits even if they had spent considerably more than the 16% of the day moving estimated by Bettridge (2010). Fully furred animals would have been thermoneutral at midday providing they did not spend more than 30% of their day moving (the maximum observed in both contemporary baboons (Bettridge, 2010) and chimpanzees [Lehmann et al., 2007]), and hairless ones would always have been thermoneutral (SOM Fig. S3). This spare thermal load capacity would have allowed sufficient extra capacity for the modest amounts of heat generated during the day by the act of feeding and by grooming behaviours that have been ignored in the model. This is important, because it means that omission of these additional thermal load costs from the model is not sufficient to negate the main findings.

## 4 Discussion

Ruxton and Wilkinson, 2011a, Ruxton and Wilkinson, 2011b suggested that the internal heat production due to movement during the day would make it impossible for bipedality to have been a response to moving out into more open wooded habitats, assuming that (a) the hominins were fully hair covered at the time they entered the savannah and (b) they were moving 100% of the time. We confirm that this result is correct, given the assumptions of the original RW model. In this respect, Ruxton and Wilkinson, 2011a, Ruxton and Wilkinson, 2011b introduced an important modification to Wheeler, 1984, Wheeler, 1990, Wheeler, 1991a, Wheeler, 1991b, Wheeler, 1992 heat load model of early australopiths. However, we suggest that, in doing so, they made several unrealistic assumptions that led them to conclude prematurely that Wheeler was wrong in concluding that bipedality yielded significant heat load savings. We identified two particularly important ones: that animals are continuously active throughout the day, and that australopiths lived at sea level. Correcting their model for these assumptions suggests that not only would the australopiths have been able to maintain their heat load within reasonable limits, but, more importantly, there would always have been a consistent, if modest, advantage to being bipedal under these conditions, whether or not they were furred.

**Figure 6:**
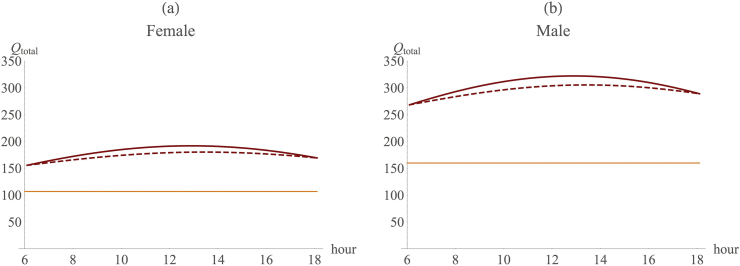
Overheating when there is no direct solar radiation at all (as would be the case in closed forest) in the case of a quadrupedal, hair covered individual. X-axis: time of day; y-axis: total heat load (*Q*_*total*_). Continuous red line: RW baseline; dashed red line: zero solar radiation case; orange: RW heat dissipation limit. (For interpretation of the references to colour in this figure legend, the reader is referred to the web version of this article.)

In fact, with the assumptions they make, the Ruxton–Wilkinson model implies that even chimpanzees would be in heat overload if they moved all the time, and so should not be able to survive even now. Since this is clearly not the case (Fig. 6), it should alert us to the fact that there is a problem with the model. In fact, in terms of thermal regime, the habitats where chimpanzees live turn out to be very similar in respect of their thermal conditions to those occupied by the australopiths (Fig. 1B), and chimpanzees in fact spend only ∼17% of the day moving (Lehmann et al., 2007). As it happens, travel time is the main constraint on great ape biogeography (Lehmann et al., 2007; also see SOM Fig. S2), and great apes pursue behavioural strategies (such as fission-fusion sociality) that allow them to reduce travel time to around 15–20% of the day (Lehmann et al., 2010). Even so, most chimpanzee populations are on the edge of survival (Lehmann et al., 2007, Lehmann et al., 2010, Lehmann and Dunbar, 2009). Given this, it is small wonder that the assumptions made by Ruxton and Wilkinson made it difficult for australopiths to survive.

Correcting the RW model for altitude and amount of time spent moving indicates that there is no thermal advantage to being bipedal in a completely shaded habitat such as forest (SOM Fig. S1). This is in striking contrast to more open habitats where there is a substantial (>10%) advantage to being bipedal. Since most real world selective advantages are in fact around 5–10% (Kingsolver et al., 2001), an advantage of this magnitude is clearly significant.

It is important to be clear that we are not suggesting that australopiths occupied open savannah grasslands. The palaeoenvironmental evidence indicates that they occupied a range of ecotone habitats that included wooded grasslands, open woodland, gallery forest and open riverine/lacustrine floodplains (Harris, 1991, Reed, 1997, White et al., 2006, Copeland et al., 2011; see also White et al., 2009, Bedaso et al., 2013) – in many ways, not dissimilar to the habitats preferred by *Papio* baboons today (Bettridge, 2010). The C_4_-pathway underground storage organ (USO) diet (with or without termites) that seems to have formed the foundation of the australopith diet (Sponheimer and Lee-Thorp, 2003, Sponheimer et al., 2005, Ungar et al., 2006, Cerling et al., 2011, Ungar and Sponheimer, 2011) is predominantly associated with the relatively open floodplains that border large rivers and lakesides. These kinds of habitats will have exposed australopiths to moderate to high levels of direct incident radiation. Even the wooded parts of these habitats are far from being shaded and are quite unlike closed forest, although they often do provide the same kind of rich ‘supermarket’ feeding patches such as fruiting figs (*Ficus* spp.) that forests do. Of more importance is the fact that travel between gallery forest night time refuges and the relatively open floodplains that provided access to USOs would have unavoidably exposed them to direct sunlight for significant parts of the day.

Our results also suggest that, with more appropriate parameterisation, the Ruxton–Wilkinson model provides an important novel finding: it offers an explanation for why australopiths typically apparently did not live at low altitudes or in coastal environments, as indeed is still the case for chimpanzees (see Lehmann et al., 2007, Lehmann and Dunbar, 2009). Surprisingly, perhaps, the ecological significance of this seems not to have been noticed: it would have made it difficult for australopiths to leave Africa, as they may have found it difficult to cope with the increased heat load in coastal habitats. The claim that australopiths did not live below ∼1000 m asl is open to empirical testing: if australopith fossils are ever found significantly below this altitude in the future, it would disprove the findings of the model and imply that some other important factor has been overlooked.

We are not, of course, necessarily suggesting that thermal savings from bipedalism are the reason why bipedalism first evolved in the hominin lineage. Our claim holds even if a form of bipedalism first emerged in the pre-australopith lineage(s) inhabiting more heavily forested environments in connection with food gathering (e.g. Thorpe et al., 2007, Crompton et al., 2008). It is important to distinguish between the factor(s) that selected for the adoption of competent, albeit imperfect, bipedal locomotion in trees (associated with a foot and lower limb still capable of efficient arboreal movement, as in *Ardipithecus* [Lovejoy et al., 2009]) and the factor(s) that selected for a subsequent radical shift in foot structure that allowed the kind of more efficient plantar bipedal locomotion found in the australopiths and later *Homo*. Our concern has been with this second step. Our claim would be that if partial bipedalism provided the australopiths with a window of ecological opportunity that made it possible for them to invade novel terrestrial habitats, thermoregulation is likely to have provided further significant selection for efficient bipedal locomotion, resulting in the final transition into *Homo*-style skeletal adaptations.

Given this, we should perhaps still ask whether bipedalism conferred other advantages sufficient on their own to promote this second phase. One possibility is that bipedalism offers substantial efficiency savings for long distance travel in long legged hominins (genus *Homo*; Pontzer et al., 2009). Bipedal locomotion is quite inefficient for chimpanzees due to their bent-hip/bent-knee posture: they consume ∼25% more energy when walking bipedally than they do when walking quadrupedally (Sockol et al., 2007). Nonetheless, only a modest change in limb length and/or muscle fascicle length would have been sufficient to make hominin bipedalism more energy efficient than chimpanzee quadrupedalism (Sockol et al., 2007). Consequently, there might well have been an added efficiency benefit to bipedalism if the australopiths were making short forays out onto more open areas beyond the lacustrine/riverine gallery forests in search of new food sources (such as USOs). However, early australopiths, at least, were markedly less efficient bipedal walkers than modern humans (Pontzer et al., 2009), and, while they must have gained some advantage, it is unlikely that this benefit on its own would have been sufficient to have provided the selection pressure for the final transition to a fully bipedal stance, especially bearing in mind that time amount of time for which this benefit would have been gained is quite small. Even allowing for australopith bipedalism being 25% more efficient than that of chimpanzees (the value pertaining to modern humans), the net energy gain for spending only 16% of the time travelling would only about to ∼4% – considerably less than the >10% benefit from thermoregulation.

An alternative possibility that has been suggested is that bipedalism offers advantages in terms of food (or tool) carrying, since this has been observed in chimpanzees (Hunt, 1994, Carvalho et al., 2012). However, chimpanzees typically do this only when crop raiding and subject to intense threat from humans; moreover, it typically involves cultivars like maize cobs, and cassava or large fruits (such as papaya) that can be gathered and carried relatively easily. Given the archaeological and trace element evidence that australopiths developed a specialization for USOs (Sponheimer and Lee-Thorp, 2003, Sponheimer et al., 2005, Ungar et al., 2006, Ungar and Sponheimer, 2011), and these are not the kind of foods that can be grabbed up in armfuls to carry away when under threat, it is difficult to see this being sufficiently advantageous to provide the needed selection pressure. Such benefits are certainly an advantage, but they are more likely to have accrued as a consequence of bipedalism rather than its cause.

On balance, then, a combination of energy savings with thermal benefits and locomotor advantages would seem to provide the most likely selection pressures favouring bipedalism in the australopiths, with the locomotor advantages probably becoming increasingly important with *Homo* in order to facilitate a more nomadic ranging pattern and the occupation of lower altitude habitats under significantly cooler thermal regimes.

Our results suggest that, while hair loss would have provided australopiths with substantial thermal advantages in more open habitats, the night time costs of reduced fur cover were very considerable and thus likely to militate against it so long as the australopiths occupied moderately high altitude habitats. Hairlessness would seem to have necessitated strategies to counteract overnight cooling and/or the occupation of lower altitude habitats. Heat loss at night can be reduced by the use of caves (which can raise mean ambient temperatures by as much as 4 °C [Barrett et al., 2004, Dunbar and Shi, 2013]) or by the regular use of fire. Although there is evidence of occasional use of fire from around 1 Ma (Gowlett et al., 1981, Goren-Inbar et al., 2004), and indirect evidence of fire use 1.9 million years ago (Wrangham et al., 1999), there is in fact little direct evidence for habitual fire use prior to ∼400 thousand years ago (ka) (Roebroeks and Villa, 2011, Dunbar and Gowlett, 2014, Shimelmitz et al., 2014), and there is no evidence at all that any australopith populations ever made use of fire. Although caves probably have been used as night time refuges intermittently throughout hominin evolution, the use of caves may not have become a regular feature until hominins developed home bases, and that may have coincided with control over fire (Shimelmitz et al., 2014) and the acquisition of a more human-like life history (Dean et al., 2001, Martin-Gonzalez et al., 2012) around 500 ka, with both being particularly associated with the occupation of high latitudes.

A more plausible suggestion is that hair loss appeared with the arrival of *Homo* around 2.0 Ma, once the climate cooling that set in after 2.5 Ma (Demenocal, 1995, Bartoli et al., 2005) allowed hominins to occupy somewhat lower altitude habitats. It is doubtful that australopiths were sufficiently mobile to make hair loss advantageous, but the appearance of a genus with a body shape better adapted to long distance travel (*Homo ergaster* locomotion was ∼50% more efficient energetically than that of early australopiths [Steudel-Numbers, 2006, Pontzer et al., 2009]), combined with the first uncontroversial evidence for the occupation of lower altitude (including coastal) habitats (as evidenced by the fact that *H. ergaster* was able to migrate out of Africa into Eurasia quite soon after its first appearance), might signal the appearance of a suite of adaptations enabling greater mobility in more open, hotter habitats. Hair loss may thus be a peculiarity of our genus, and may have played a small but important role in allowing *Homo* to escape the confines of Africa.

## Appendix

### The formal model

We go through the RW model in detail here because we found that the descriptions given by Ruxton and Wilkinson, 2011a, Ruxton and Wilkinson, 2011b are not always clear or explicit. In addition, there appears to be an error in their description of the model (though not, we believe, in their implementation of the model).

Formally, the following equations define the system.

#### Thermal environment

The air temperature that an animal is facing, $T_{a}$, is a ratio between the ground temperature, $T_{g}$, and temperature at 2 m height, $T_{200}$, dependent on the height and posture of the animal. Thus the temperature at 2 m above ground is:

(1) $$T_{200}\left(t\right)=\tau_{200}-25+25\;\text{Sin}\left(\frac{\pi \left(t-5\right)}{18}\right)\text{.}$$

where $\tau_{200}$ is the maximum daily temperature in Celsius, and time *t* ranges from 0 to 24 h.

The temperature at ground level is:

(2) $$T_{g}\left(t\right)=\tau_{200}+5-35+35\;\text{Sin}\left(\frac{\pi \left(t-5\right)}{16}\right)\text{.}$$

Given the temperature at ground level, and 2 m above ground, the actual temperature faced by the animal (assuming that it is shorter than 2 m) is:

(3) $$T_{a}=T_{200}+\alpha \left(T_{g}-T_{200}\right)\text{.}$$

where α is dependent on the posture and height of the animal, with higher values of α corresponding to lower characteristic height.

The air temperature that the animal is facing will be moderated by the speed of air around the body. The RW model assumes that this is due purely to the movement of the body, which is dependent on the leg length, *L*. Thus the speed of the movement of the body while walking (and thus, also that of the movement of the air) in m/s, *v*, is:

(4) $$v=1.7\sqrt{L}\text{.}$$

#### Radiation environment

The model needs to account for both short and long wave radiation hitting the body surface.

The direct short wave radiation, *S*, is:

(5) $$S=865\;\text{Sin}\left(\frac{\pi \left(t-6\right)}{12}\right)\text{.}$$

where we assume that the function is evaluated only at day time on the equator: 6≤t≤18.

The indirect diffuse irradiance, *s*, from the solar irradiance scattering in the atmosphere is 10% of the total short wave radiation:

(6) $$s=\frac{S}{9}\text{.}$$

The long wave background radiation from the sky is:

(7) $$R_{sky}=213+5.5T_{200}\text{.}$$

and that by reflectance from the ground is:

(8) $$R_{g}=\sigma {\left(T_{g}+273\right)}^{4}\text{.}$$

where σ is the Stefan–Boltzmann constant (5.67·10^−8^ W m^−^^2^ K^−^^4^).

#### Total exposure to radiation

The RW model assumes that for each point of the body surface there are four possibilities: it may or may not be directly exposed to solar radiation, and it may or may not be covered by hair.

The model assumes that the average background radiation is the same for the dorsal (facing towards the sun) and ventral (facing towards the ground) parts of the body. (We do not consider the question of whether this assumption is justified because we want to keep our analysis as close to the RW model as possible, but we think any deviations away from equality are likely to have only a modest effect.) Assuming that half of the body is exposed to solar radiation, this means that the ventral part of the body that is not exposed to solar radiation (denoted as *vent*), receives the following total amount of heat:

(9) $$Q_{inc.vent}=0.5\left(s+r\left(S+s\right)+R_{sky}+R_{\text{g}}\right)\text{.}$$

where *r* is surface reflectivity assumed to be 15%. This implies that the dorsal parts of the body that are exposed to direct solar radiation (denoted as *dors*) receive the following amount of heat:

(10) $$Q_{inc.dors}=S+Q_{inc.vent}\text{.}$$

We assume that some parts of the body are covered in thick hair, which absorbs some of the short wave energy. This alters (9) and (10) in the following way:

(11) $$Q_{abs.vent}=0.5\left(as+ar\left(S+s\right)+R_{sky}+R_{\text{g}}\right)\text{.}$$

(12) $$Q_{abs.dors}=aS+Q_{abs.vent}\text{.}$$

where *a* is the proportion of short wave energy absorbed by the coat (assumed to be 80%).

The four possible states of each point of the body, thus yield four different radiation volumes: *Q*_*inc.vent*_ (no hair, no direct solar radiation), *Q*_*inc.dors*_ (no hair, direct solar radiation), *Q*_*abs.vent*_ (hair covered, no direct solar radiation), and *Q*_*abs.dors*_ (hair covered, direct solar radiation).

For each of these, the total net heat gain from the environment can be computed when corrected with the radiation reflected from the surface of either the skin or the hair (implicitly assumed to be identical in the RW model), and the effect of the air moving around the body:

(13) $$Q_{gain}=Q_{abs}-\left(\sigma {\left(T_{s}+273\right)}^{4}+k\sqrt{v}\left(T_{s}-T_{a}\right)\right)\text{.}$$

where the convection coefficient, *k*, is $9.8W/m^{2{}^{\circ}}C$, and *v* is the velocity of the air defined in Eq. (4), and $T_{s}$ is the surface temperature (unknown at this point).

To find the surface temperature, $T_{s}$, the model assumes that this gain is the equivalent to the heat that travels from the surface to the skin

(14) $$C\left(T_{s}-T_{c}\right)=Q_{abs}-\left(\sigma {\left(T_{s}+273\right)}^{4}+k\sqrt{v}\left(T_{s}-T_{a}\right)\right)\text{.}$$

where $T_{c}$ is the skin temperature, assumed to be 37 °C, and *C* is the thermal conductance of the pelt.

(Note that in the formulation given in the original papers [Ruxton and Wilkinson, 2011a, Ruxton and Wilkinson, 2011b], this equation contained an error: they give $T_{s}^{4}$ rather than ${\left(T_{s}+273\right)}^{4}$ for the radiation reflected from the surface.)

Equation (14) yields four solutions for $T_{s}$, of which only one is positive and real, given the parameter range used in the model. We assume that this is a unique solution, and substitute it back into (13) to evaluate the net heat gain in the four net radiation states: *Q*_*gain.inc.vent*_,*Q*_*gain.inc.dors*_,*Q*_*gain.abs.vent*_,*Q*_*gain.abs.dors*_.

To calculate the total environmental heat load on the body, *Q*_*env*_, we need to know the total surface of the body, *A*, as well as the portion of the surface that receives each of the four types of radiation.

For the surface, we assume, in line with RW, that

(15) $$A=0.11M^{0.67}\text{.}$$

where *M* is the mass of the body in kg.

Thus, the total environmental heat load is:

(16) $$Q_{env}=\rho \left(\beta Q_{gain.abs.dors}+\left(1-\beta \right)Q_{gain.abs.vent}\right)+\left(1-\rho \right)\left(\beta Q_{gain.inc.dors}+\left(1-\beta \right)Q_{gain.inc.vent}\right)\text{.}$$

where ρ is the proportion of the body covered by hair, and β is the proportion of the body that is exposed to direct solar radiation. In line with RW, we assume that β is dependent on whether the animal is biped or quadruped in the following way:

(17) $$\beta =\{\begin{array}{l} 0.23-0.15\;\mathrm{Sin}\left(\frac{\pi \left(t-6\right)}{12}\right)\;\text{if}\;\text{biped}\\ 0.21-0.03\;\text{Sin}\left(\frac{\pi \left(t-6\right)}{12}\right)\;\text{if}\;\text{quadruped} \end{array}\text{.}$$

Assuming again that the function is evaluated only at day time on the equator (i.e. where 6≤t≤18).

#### Internal metabolic heat load

In line with RW, we assume that the animal produces a basic level of metabolic heat, to which the heat generated by moving the body (with the speed defined in Eq. (4)) is added.

(18) $$Q_{met}=0.9\left(6.03M^{0.7}+10.7vM^{0.68}\right)\text{.}$$

where we assumed that 10% of the heat is lost through respiration.

Then the total heat load is the sum of the environmental and metabolic heat loads:

(19) $$Q_{total}=Q_{env}+Q_{met}\text{.}$$

Equations (1), (2), (5), (6), (7), (8), (9), (10), (11), (12), (13), (14), (15), (16), (17), (18), (19)) define the system for the RW version of Wheeler, 1984, Wheeler, 1990, Wheeler, 1991a, Wheeler, 1991b, Wheeler, 1992 heat load model, represented by the red lines in the four panels of Figure 2.

## References

[bib1] Aiello L.C., Wells J.C.K. (2002). Energetics and the evolution of the genus *Homo*. Ann. Rev. Anthropol..

[bib2] Alemseged Z., Geraads D. (1998). *Theropithecus atlanticus* (Thomas, 1884) (Primates: Cercopithecidae) from the late Pliocene of Ahl al Oughlam, Casablanca, Morocco. J. Hum. Evol..

[bib3] Barrett L., Gaynor D., Rendall D., Mitchell D., Henzi S.P. (2004). Habitual cave use and thermoregulation in chacma baboons (Papio hamadryas ursinus). J. Hum. Evol..

[bib4] Bartoli G., Sarnthein M., Weinelt M., Erlenkeuser H., Garbe-Schonberg D., Lea D.W. (2005). Final closure of Panama and the onset of northern hemisphere glaciation. Earth Planet Sci. Lett..

[bib5] Bedaso Z.K., Wynn J.G., Alemseged Z., Geraads D. (2013). Dietary and paleoenvironmental reconstruction using stable isotopes of herbivore tooth enamel from middle Pliocene Dikika, Ethiopia: Implication for *Australopithecus afarensis* habitat and food resources. J. Hum. Evol..

[bib6] Bettridge C.M. (2010). Reconstructing australopithecine socioecology: strategic modelling based on modern primates.

[bib7] Bettridge C.M., Dunbar R.I.M. (2012). Modeling the Biogeography of fossil baboons. Int. J. Primatol..

[bib8] Bettridge C.M., Lehmann J., Dunbar R.I.M. (2010). Trade-offs between time, predation risk and life history, and their implications for biogeography: A systems modelling approach with a primate case study. Ecol. Modell..

[bib9] Biberson P. (1961). Le Paléolithique inférieure du Maroc Altalntique.

[bib10] Bibi F. (2007). Origin, paleoecology, and paleobiogeography of early Bovini. Palaeogeogr. Palaeocl..

[bib11] Bocherens H., Sandrock O., Kullmer O., Schrenk F. (2011). Hominin palaeoecology in Late Pliocene Malawi: First insights from isotopes (^13^C, ^18^O) in mammal teeth. S. Afr. J. Sci..

[bib13] Boaz N.T., El-Arnauti A., Augusti J., Bernor R.L., Pavlakis P.P., Rook L. (2008). Temporal, lithographic, and biochronologic setting of the Sahabi formation, north-central Libya. Geol. E. Libya.

[bib14] Bonnefille R., Vincens A., Buchet G. (1987). Palynology, stratigraphy and palaeoenvironment of a Pliocene hominid site (2.9-3.3 M.Y.) at Hadar, Ethiopia. Palaeogeogr. Palaeocl..

[bib15] Brunet M., Guy F., Pilbeam D., Mackaye H.T., Likius A., Ahounta D., Beauvilain A., Blondel C., Bocherens H., Boisserie J.R., De Bonis L., Coppens Y., Dejax J., Denys C., Duringer P., Eisenmann V.R., Fanone G., Fronty P., Geraads D., Lehmann T., Lihoreau F., Louchart A., Mahamat A., Merceron G., Mouchelin G., Otero O., Campomanes P.P., De Leon M.P., Rage J.C., Sapanet M., Schuster M., Sudre J., Tassy P., Valentin X., Vignaud P., Viriot L., Zazzo A., Zollikofer C. (2002). A new hominid from the Upper Miocene of Chad, central Africa. Nature.

[bib16] Carvalho S., Biro D., Cunha E., Hockings K., McGrew W.C., Richmond B.G., Matsuzawa T. (2012). Chimpanzee carrying behaviour and the origins of human bipedality. Curr. Biol..

[bib17] Cerling T.E., Mbua E., Kirera F.M., Manthi F.K., Grine F.E., Leakey M.G., Sponheimer M., Uno K.T. (2011). Diet of *Paranthropus boisei* in the early Pleistocene of East Africa. Proc. Natl. Acad. Sci. USA.

[bib18] Copeland S.R., Sponheimer M., de Ruiter D.J., Lee-Thorp J.A., Codron D., le Roux P.J., Grimes V., Richards M.P. (2011). Strontium isotope evidence for landscape use by early hominins. Nature.

[bib19] Crompton R.H., Vereecke E.E., Thorpe S.K.S. (2008). Locomotion and posture from the common hominoid ancestor to fully modern hominins, with special reference to the last common panin/hominin ancestor. J. Anat..

[bib20] Dean C., Leakey M.G., Reid D., Schrenk F., Schwartz G.T., Stringer C., Walker A. (2001). Growth processes in teeth distinguish modern humans from *Homo erectus* and earlier hominins. Nature.

[bib21] Demenocal P.B. (1995). Pliopleistocene African climate. Science.

[bib22] Dowsett H., Barron J., Poore R., Thompson R., Cronin T., Ishman S., Willard D. (1999). Middle Pliocene Palaeoenvironmental Reconstruction: PRISM2, US Geological Survey Open-File report.

[bib23] Dunbar R.I.M. (1979). Energetics, thermoregulation and the behavioral ecology of Klipspringer. Afr. J. Ecol..

[bib24] Dunbar R.I.M. (1998). Impact of global warming on the distribution and survival of the gelada baboon: a modelling approach. Global Change Biol..

[bib25] Dunbar R.I.M., Gowlett J.A.J., Dunbar R.I.M., Gamble C., Gowlett J.A.J. (2014). Fireside chat: the impact of fire on hominin socioecology. Lucy to Language: the Benchmark Papers.

[bib26] Dunbar R.I.M., Shi J.B. (2013). Time as a constraint on the distribution of feral goats at high latitudes. Oikos.

[bib27] Dunbar R.I.M., Korstjens A.H., Lehmann J. (2009). Time as an ecological constraint. Biol. Rev..

[bib28] Frost F.R., Kullmer O. (2008). Cercopithecidae from the Pliocene Chiwondo beds, Malawi-rift. Geobios.

[bib29] Gebremedhin K.G., Wu B.X. (2001). A model of evaporative cooling of wet skin surface and fur layer. J. Therm. Biol..

[bib30] Geraads D., Hublin J.-J., Jaeger J.-J., Tong H., Sen S., Toubeau P. (2008). The Pleistocene hominid site of Ternifine, Algeria: New results on the environment, age, and human industries. Quaternary Research.

[bib31] Goren-Inbar N., Alperson N., Kislev M.E., Simchoni O., Melamed Y., Ben-Nun A., Werker E. (2004). Evidence of hominin control of fire at Gesher Benot Ya'aqov, Israel. Science.

[bib32] Gowlett J.A.J., Harris J.W.K., Walton D., Wood B.A. (1981). Early archaeological sites, hominid remains and traces of fire from Chesowanja, Kenya. Nature.

[bib33] Harris J.M., Harris J.M. (1991). Family Giraffidae. Koobi Fora Research Project. The Fossil Ungulates: Geology, Fossil Artiodactyls, and Palaeoenviron- ments.

[bib34] Hendey Q.B. (1981). Palaeoecology of the Late tertiary fossil occurrences in ‘E’ quarry, Langebaanweg, South Africa, and a reinterpretation of their geological context. Ann. S. Afr. Mus..

[bib35] Hiley P.G. (1976). The thermoregulatory responses of the galago (*Galago crassicaudatus*), the baboon (*Papio cynocephalus*) and the chimpanzee (*Pan satyrus*) to heat stress. J. Physiol..

[bib36] Hill R.A. (2006). Thermal constraints on activity scheduling and habitat choice in baboons. Am. J. Phys. Anthropol..

[bib37] Hunt K.D. (1994). The evolution of human bipedality: ecology and functional morphology. J. Hum. Evol..

[bib38] Jacobson M.Z. (2005). Fundamentals of Atmospheric Modelling.

[bib39] Kappelman J., Alçiçek M.C., Kazanci N., Schultz M., Ôzkul M., Sen S. (2008). First *Homo erectus* from Turkey and implications for migrations into temperate Eurasia. Amer. J. Phys. Anthrop..

[bib40] Kingsolver J.G., Hoekstra H.E., Hoekstra J.M., Berrigan D., Vignieri S.N., Hill C.E., Hoang A., Gibert P., Beerli P. (2001). The strength of phenotypic selection in natural populations. Am. Nat..

[bib41] Lehmann J., Dunbar R. (2009). Implications of body mass and predation for ape social system and biogeographical distribution. Oikos.

[bib42] Lehmann J., Korstjens A.H., Dunbar R.I.M. (2007). Fission-fusion social systems as a strategy for coping with ecological constraints: a primate case. Evol. Ecol..

[bib43] Lehmann J., Korstjens A.H., Dunbar R.I.M. (2010). Apes in a changing world – the effects of global warming on the behaviour and distribution of African apes. J. Biogeogr..

[bib44] Lovejoy C.O., Suwa G., Spurlock L., Asfaw B., White T.D. (2009). The pelvis and femur of *Ardipithecus ramidus*: The emergence of upright walking. Science.

[bib45] Maloiy G.M.O., Rugangazi B.M., Clemens E.T. (1988). Physiology of the dik-dik antelope. Comp. Biochem. Physiol. A Physiol..

[bib46] Martin-Gonzalez J.A., Mateos A., Goikoetxea I., Leonard W.R., Rodriguez J. (2012). Differences between Neandertal and modern human infant and child growth models. J. Hum. Evol..

[bib47] Mount L.E. (1979). Adaptation to Thermal Environment: Man and his Productive Animals.

[bib48] NOAA (2014). National Climate Data Center. http://www.ncdc.noaa.gov.

[bib49] Partridge T.C., Ruddiman W.F. (1997). Late Neogene uplift in Eastern and Southern Africa and its paleoclimatic implications. Tectonic Uplift and Climate Change.

[bib50] Pickford M., Senut B., Gommery D., Treil J. (2002). Bipedalism in *Orrorin tugenensis* revealed by its femora. C. R. Palevol.

[bib51] Pontzer H., Raichlen D.A., Sockol M.D. (2009). The metabolic cost of walking in humans, chimpanzees, and early hominins. J. Hum. Evol..

[bib52] Precht H., Brück K. (1973). Temperature and Life.

[bib53] Reed K.E. (1997). Early hominid evolution and ecological change through the African Plio-Pleistocene. J. Hum. Evol..

[bib54] Roberts S.C., Dunbar R.I.M. (1991). Climatic influences on the behavioral ecology of chanler mountain reedbuck in Kenya. Afr. J. Ecol..

[bib55] Robinson M., Doswett H.J., Chandler M.A. (2008). Pliocene role in assessing future climate impacts. Eos Transactions.

[bib56] Roebroeks W., Villa P. (2011). On the earliest evidence for habitual use of fire in Europe. Proc. Natl. Acad. Sci. USA.

[bib57] Ruxton G.D., Wilkinson D.M. (2011). Avoidance of overheating and selection for both hair loss and bipedality in hominins. Proc. Natl. Acad. Sci. USA.

[bib58] Ruxton G.D., Wilkinson D.M. (2011). Thermoregulation and endurance running in extinct hominins: Wheeler's models revisited. J. Hum. Evol..

[bib59] Sahnouni M., Toth N., Schick K. (2006). The North African Early Stone Age and the sites at Ain Hanech, Algeria. The Olduwan: Case Studies into the Earliest Stone Age, pp. 77–111.

[bib60] Sandrock O., Kullmer O., Schrenk F., Juwayeyi Y.M., Bromage T.G., Bobe R., Alemseged Z., Behrensmeyer A.K. (2007). Fauna, taphonomy, and ecology of the Plio-Pleistocene Chiwondo Beds, Northern Malawi. Hominin Environments in the East African Pliocene: An Assessment of the Faunal Evidence.

[bib61] Shimelmitz R., Kuhn S.L., Jelinek A.J., Ronen A., Clark A.E., Weinstein-Evron M. (2014). ‘Fire at will’: The emergence of habitual fire use 350,000 years ago. J. Hum. Evol..

[bib62] Sockol M.D., Raichlen D.A., Pontzer H. (2007). Chimpanzee locomotor energetics and the origin of human bipedalism. Proc. Natl. Acad. Sci. USA.

[bib63] Sponheimer M., Lee-Thorp J.A. (2003). Differential resource utilization by extant great apes and australopithecines: towards solving the C-4 conundrum. Comp. Biochem. Physiol. A Mol. Integr. Physiol..

[bib64] Sponheimer M., Lee-Thorp J., de Ruiter D., Codron D., Codron J., Baugh A.T., Thackeray F. (2005). Hominins, sedges, and termites: new carbon isotope data from the Sterkfontein valley and Kruger National Park. J. Hum. Evol..

[bib65] Steudel-Numbers K.L. (2006). Energetics in *Homo erectus* and other early hominins: The consequences of increased lower-limb length. J. Hum. Evol..

[bib66] Thorpe S.K.S., Holder R.L., Crompton R.H. (2007). Origin of human bipedalism as an adaptation for locomotion on flexible branches. Science.

[bib67] Ungar P.S., Sponheimer M. (2011). The diets of early hominins. Science.

[bib68] Ungar P.S., Grine F.E., Teaford M.F. (2006). Diet in early *Homo*: A review of the evidence and a new model of adaptive versatility. Annu. Rev. Anthropol..

[bib69] Wheeler P.E. (1984). The evolution of bipedality and loss of functional body hair in hominids. J. Hum. Evol..

[bib70] Wheeler P.E. (1990). The influence of thermoregulatory selection pressures on hominid evolution. Behav. Brain. Sci..

[bib71] Wheeler P.E. (1991). The influence of bipedalism on the energy and water budgets of early hominids. J. Hum. Evol..

[bib72] Wheeler P.E. (1991). The thermoregulatory advantages of hominid bipedalism in open equatorial environments - the contribution of increased convective heat-loss and cutaneous evaporative cooling. J. Hum. Evol..

[bib73] Wheeler P.E. (1992). The thermoregulatory advantages of large body size for hominids foraging in savanna environments. J. Hum. Evol..

[bib74] White T.D., WoldeGabriel G., Asfaw B., Ambrose S., Beyene Y., Bernor R.L., Boisserie J.R., Currie B., Gilbert H., Haile-Selassie Y., Hart W.K., Hlusko L.J., Howell F.C., Kono R.T., Lehmann T., Louchart A., Lovejoy C.O., Renne P.R., Saegusa H., Vrba E.S., Wesselman H., Suwa G. (2006). Asa Issie, aramis and the origin of *Australopithecus*. Nature.

[bib75] White T.D., Ambrose S.H., Suwa G., Su D.F., DeGusta D., Bernor R.L., Boisserie J.R., Brunet M., Delson E., Frost S., Garcia N., Giaourtsakis I.X., Haile-Selassie Y., Howell F.C., Lehmann T., Likius A., Pehlevan C., Saegusa H., Semprebon G., Teaford M., Vrba E. (2009). Macrovertebrate paleontology and the Pliocene habitat of *Ardipithecus ramidus*. Science.

[bib76] Wrangham R.W., Jones J.H., Laden G., Pilbeam D., Conklin-Brittain N. (1999). The raw and the stolen – Cooking and the ecology of human origins. Curr. Anthropol..

