# Supplementary material for: Bipedality and hair loss in human evolution revisited: The impact of altitude and activity scheduling
Source: J Hum Evol. 2016 May;94:72–82. doi: 10.1016/j.jhevol.2016.02.006 (PMC4874949; doi:10.1016/j.jhevol.2016.02.006)
Supplement: Supplementary file 1 [file mmc1.docx]

**Supplementary Online Material for**

**Bipedality and Hairloss in Human Evolution Revisited:**

**The Impact of Altitude and Activity Scheduling**

**Tamás Dávid-Barrett** and **Robin I.M. Dunbar**

**Fossil sites**

We use the climate data for fossil australopith sites from Betteridge & Dunbar (2012). The sites, the matched contemporary site from which climate was estimated and the mean annual temperature for the matched site, are given in Table S1. Where possible, the matched sites are from published studies derived by fossil faunal comparison (Shipman and Harris 1988; Hernández Fernández and Vrba 2006; Reed 2008). For fossil localities where no modern day equivalent was available from the literature, we adapted the method of faunal assemblage comparison used by these authors, and matched sites for the percentage composition of each bovid tribe (calculated from the number of individual animals when necessary) for fossil and extant modern faunal assemblages. Where different datasets were available for a site, the most recent was used. Some studies differ in how they categorise bovid tribes: Reed (2008), for example, provides data on the tribe Tragelaphini, yet *Tragelaphus* spp. are included in the ‘Bovinae’ category alongside several other genera by Hernández Fernández & Vrba (2006). In such cases, it was necessary to adopt a more general classification to enable all sites to be compared in the same way. For the same reason, the percentage composition of non-bovid fauna was also discounted from Reed (2008) and Hernandez-Fernandez & Vrba (2006), as these taxa were not given by Shipman & Harris (1988). Bivariate correlations compared fossil faunal assemblages and modern assemblages, and each fossil site was then paired with the modern site whose faunal assemblage most closely and strongly correlated with its own. Although some of these matched sites are geographically near to each other, many are not and may even be in quite different part of Africa: climatic similarity is more important than co-location *per se*.

Table S1. Australopith fossil sites, with matched contemporary site and mean annual temperature

| **Country** | **Formation** | **Member/ Sub-member** | **Matched**  **modern site*** | **Temp**  **^o^C** |
| --- | --- | --- | --- | --- |
| Ethiopia | Busidima | DANA | Sennar, Sudan | 28.3 |
| Ethiopia | Busidima | MAKA | Kafue, Zambia | 21.0 |
| Ethiopia | Hadar | Basal/BM | Hluhluwe, S Africa | 26.6 |
| Ethiopia | Hadar | DD1 | Hluhluwe, S Africa | 26.6 |
| Ethiopia | Hadar | DD2 | Mupa, Angola | 26.6 |
| Ethiopia | Hadar | DD3 | Hluhluwe, S Africa | 26.6 |
| Ethiopia | Hadar | KH1 | Hluhluwe, S Africa | 26.6 |
| Ethiopia | Hadar | KH2 | Amboseli, Kenya | 26.6 |
| Ethiopia | Hadar | SH1 | Manyara, Tanzania | 26.6 |
| Ethiopia | Hadar | SH2 | Kwange, Nigeria | 26.6 |
| Ethiopia | Hadar | SH3 | Chobe, Kenya | 26.6 |
| Ethiopia | Hadar | SH4 | Hluhluwe, S Africa | 26.6 |
| Ethiopia | Hadar | SHT | Hluhluwe, S Africa | 21.6 |
| Ethiopia | Shungura | C | Hwange, Zimbabwe | 20.8 |
| Ethiopia | Shungura | B | Mupa, Angola | 28.0 |
| Ethiopia | Shungura | D | W NP, Benin | 24.0 |
| Ethiopia | Shungura | E | Kruger, S Africa | 24.0 |
| Ethiopia | Shungura | F | Hwange, Zimbabwe | 24.0 |
| Ethiopia | Shungura | LG | Hwange, Zimbabwe | 24.0 |
| Kenya | Kanapoi |  | Gaberone, Botswana | 20.2 |
| Kenya | Koobi Fora | KBS | St Floris, CAFR | 26.8 |
| Kenya | Koobi Fora | Tulu Bor/LTB | Pendjari, Burkino Faso | 25.9 |
| Kenya | Koobi Fora | Tulu Bor/UTB | Mupa, Angola | 22.7 |
| Kenya | Koobi Fora | Lokochot | W NP, Benin | 27.3 |
| Kenya | Koobi Fora | Okote | St Floris, CAFR | 26.8 |
| Kenya | Koobi Fora | Burgi/ UBU | St Floris, CAFR | 26.8 |
| Kenya | Lothagam | APK | Gaberone, Botswana | 20.2 |
| Kenya | Nachukui | Kaitio | Bicuar, Angola | 21.8 |
| Kenya | Nachukui | LLM | Omo, Ethiopia | 24.9 |
| Kenya | Nachukui | NAT | St Floris, CAFR | 26.8 |
| Kenya | Nachukui | ULL | Manyara, Tanzania | 19.7 |
| Kenya | Usno | U-12 | Hluhluwe, S Africa | 21.6 |
| Tanzania | Olduvai | Bed 1/ FLK-Zinj | Kainji, Nigeria | 27.4 |
| Tanzania | Olduvai | Bed 1/ FLKNN-3 | St Floris, CAFR | 26.8 |
| Tanzania | Olduvai | Bed 2/BK | Kainji, Nigeria | 27.4 |
| Tanzania | Olduvai | Bed 2/HWK | Serengeti, Tanzania | 20.3 |
| S Africa | Kromdraai | KA | Serengeti, Tanzania | 20.3 |
| S Africa | Kromdraai | KB | Semara, W. Sahara | 21.9 |
| S Africa | Sterkfontein | 5E-A | Omo, Ethiopia | 20.8 |
| S Africa | Swartkrans | 1 | Ngorongoro, Tanzania | 20.8 |
| S Africa | Swartkrans | 2 | Kalahari Gemsbok | 18.1 |

* Each fossil site is matched to a contemporary modern site on the basis of faunal profile. The mean annual temperature of the contemporary site provides the estimate for the fossil site. Source: Bettridge & Dunbar (2012).

**Additional model results**

In our model, we introduced three new refinements, each of which reduces the overheating effect of the baseline RW model (see Fig. 3 in main text, and Table S2).

Table S2. Percentage change to the RW model’s heat load as a consequence of introducing the three individual refinements

|  | Female  Biped  Covered | Female  Biped  Hairless | Female  Quad  Covered | Female  Quad  Hairless | Male  Biped  Covered | Male  Biped  Hairless | Male  Quad  Covered | Male  Quad  Hairless |
| --- | --- | --- | --- | --- | --- | --- | --- | --- |
| 16% average daily movement, concentrated morning and evening | -55.89 | -45.46 | -55.29 | -39.54 | -58.69 | -49.02 | -58.15 | -43.09 |
| Altitude at 1000m | -3.54 | -45.73 | -3.48 | -39.77 | -3.15 | -43.9 | -3.11 | -38.6 |
| Shade midday | -0.63 | -10.03 | -0.87 | -11.88 | -0.54 | -9.13 | -0.74 | -10.93 |

One of the main claims in the original RW paper (2011a,b) was that the thermal advantage gained from switching to bipedality from quadrupedal locomotion is negligible. However, their argument referred to absolute comparisons among the different cases. A more appropriate method would be to calculate the change in total heat load due to the switch to bipedality as a ratio of the entire daily heat load range. To do this, let us express the relative advantage of bipedality at any one time in the following way:

 (A1)

Our results show that there is approximately 5% gain from bipedality in all the cases, including the baseline RW case (Table S3).

Table S3. Daily total thermoregulatory advantage in percentage, from switching from quadrupedality to bipedality, in percentage of daily heat load balance magnitude, calculated as max – min of the energy balance during the day, see equation (A1)

|  | Female  Covered | Female  Hairless | Male  Covered | Male  Hairless |
| --- | --- | --- | --- | --- |
| RW | 5.35% | 5.52% | 5.23% | 5.38% |
| 16% average daily movement, concentrated morning and evening | 5.81% | 5.78% | 5.67% | 5.67% |
| Altitude at 1000m | 5.4% | 5.56% | 5.28% | 5.42% |
| Shade midday | 4.45% | 4.53% | 4.35% | 4.43% |

When rerunning the model without direct sunshine (as in dense forest), we found that, as expected, the pressure towards bipedality decreases (Fig. S1).


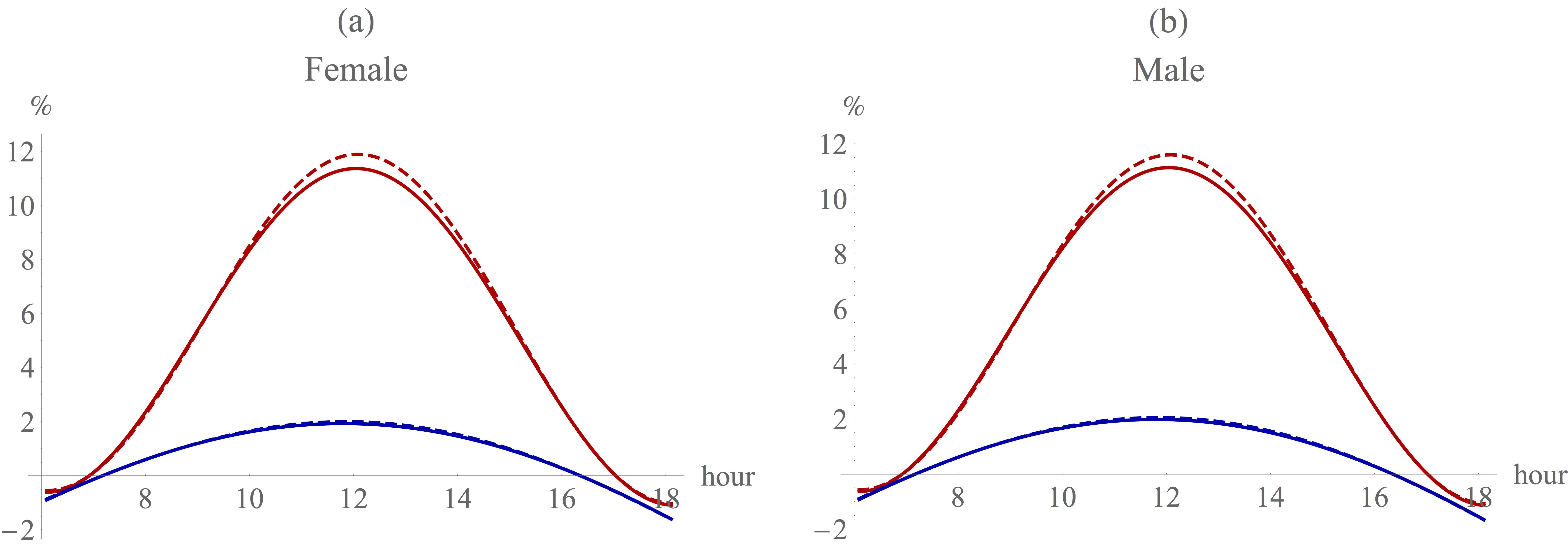


Fig. S1. The percentage advantage from switching from a quadrupedal to a bipedal stance for (a) females and (b) males, respectively, where there is no solar radiation. Red: RW model baseline (same as the red lines in Fig. 3 of the main paper), blue: zero direct solar radiation. Dash: hairless; continuous: hair covered.

We used real world 24-hour temperature profiles from three East African sites at varying altitude (NOAA, 2014) to estimate the effect of hairloss on the total daily energy need (see Fig. 5 of the main manuscript). For the altitude and latitude information see Table S4. We integrated heat load across the hours when the net heat load is negative and converted this to kcals. Table S4 suggests that there is a substantial net energy deficit due to low night time temperatures.

Table S4. The calorie deficit of hairless (15% covered) quadrupeds in three different locations, using current day temperatures from East African sites (NOAA, 2014).^a^

| Location | Nakuru | Kisumi | Lodwar |
| --- | --- | --- | --- |
| Altitude | 1850m | 1171m | 507m |
| Latitude | 0.3000 S | 0.1000 S | 3.1166 N |
| Female deficit | -3559 kcal | -2453 kcal | -1055 kcal |
| Male deficit | -5628 kcal | -3868 kcal | -1656 kcal |

^a^The deficit is calculated as the integrated total during the periods in which the net heat balance is negative. S = south; N = north; kcal = kilocalorie.

We can calculate the maximum proportion of time that could be spent moving at any particular point of the day without the animal exceeding its heat shedding limit by first evaluating the full equation set under two conditions: (1) assuming that the animal spends the entire time moving and (2) assuming no movement at all, and then calculating the maximum ratio of the movement condition such that *Q_Total_*<0. Figure S2 suggests that animals could spend as much as 50-60% of each hour moving in the early morning and late afternoon, and as much as 35-40% when moving around midday. This confirms that our results are not dependent on the specific value of 16% moving time estimated by Bettridge (2010) using a time budget model, but would still yield benefits even if this underestimated actual travel time by as much as a half (which is unlikely, given that australopith time budgets actually had no spare capacity at all [Bettridge, 2010]). It also confirms that the morning and the evening are more likely times for individuals to move about than the hot midday.


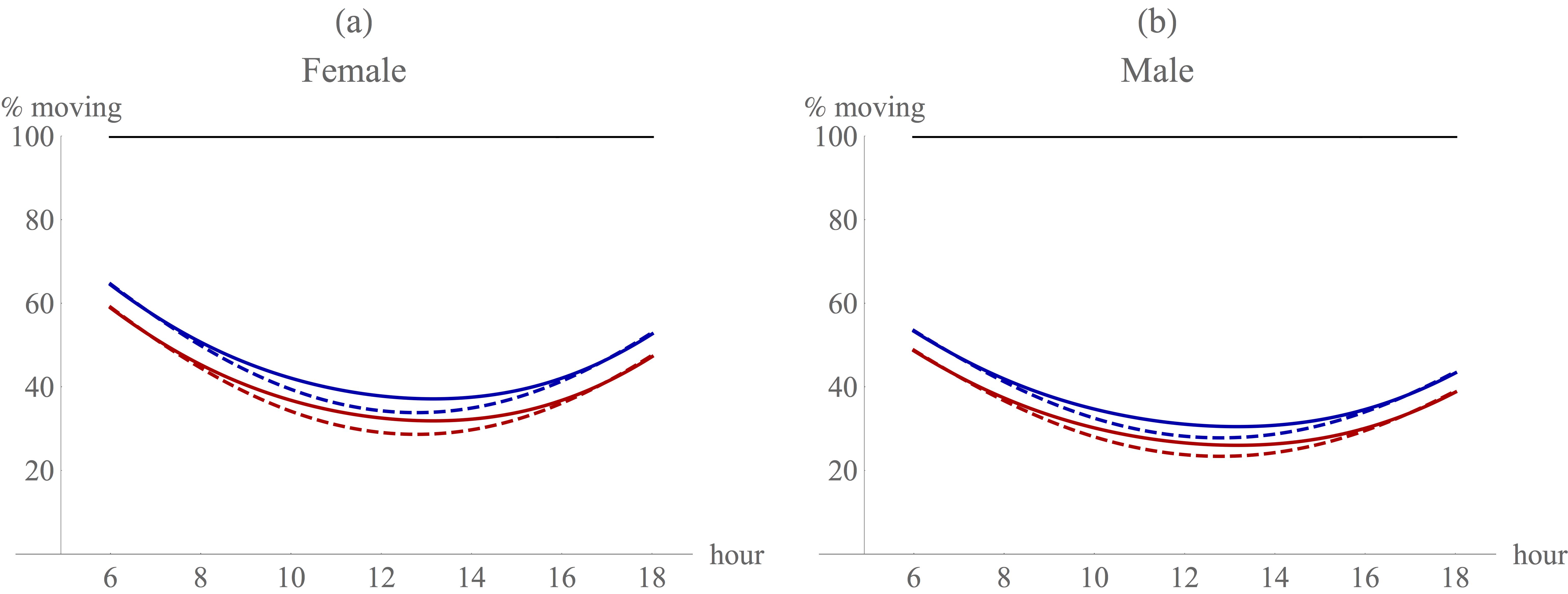


Fig. S2. Maximum percentage of each hour that could be spent moving without exceeding the limit on the animal’s capacity to shed heat by heat exchange with the environment when considering all three refinements combined. Continuous line: bipedal 100% hair cover; dashed line: quadrupedal 100% hair cover; red: at sea level; blue: at 1000 m asl. The hairless individuals can always shed enough heat, and hence their limit is at 100%, represented by a black line. The results were calculated by evaluating the model at each point in time either assuming 100% movement, or assuming 0% movement, and then calculating the weighted average of the two cases such that the net overheating remained under zero.

**Sensitivity Analyses**

We ran sensitivity analyses for the effects of the main parameters.

Fig. S3 shows the effect of varying the percentage of the day spent moving between 10% and 50% (Fig. 2 in the main text). All values result in savings in thermoregulation costs compared to the RW model. For fully furred individuals, animals are thermoneutral at midday providing they spend less than 30% of the day moving. Hairless animals are always thermoneutral, even under the assumptions of the RW model.


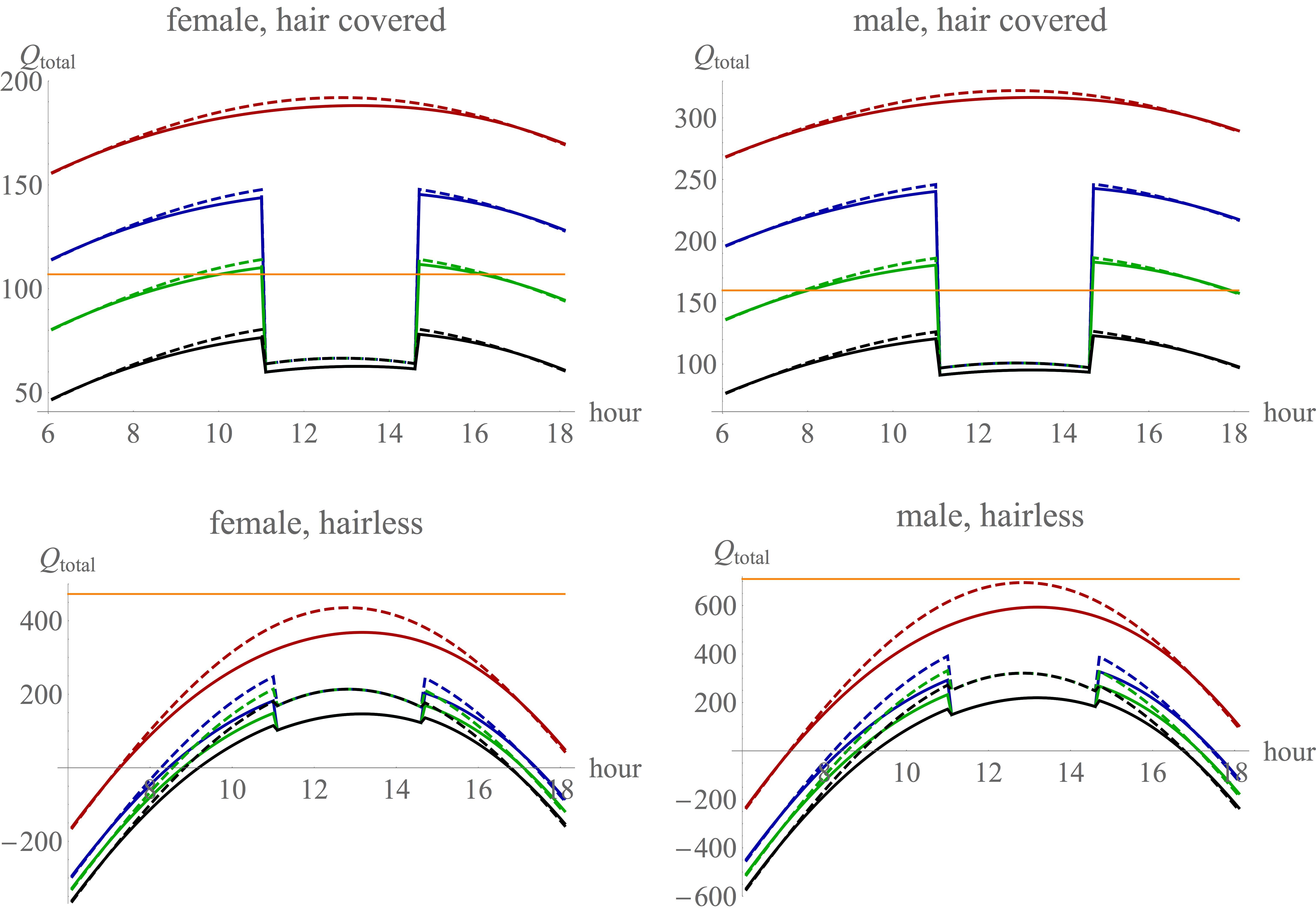


Fig. S3. Sensitivity of the results depicted in Fig. 2 of the paper to the percentage of the day spent moving, assuming a midday total rest. It is assumed in this calculation that the baseline temperature is 35.5^o^C, estimated to be associated with 1000 m asl.. Red: RW model baseline; blue, green, black: RW model with all three additional factors included; with daily average movement at 50% for blue, 30% for green, and 10% for black. Dash: quadrupedal; continuous: bipedal. Orange: heat dissipation limit.

Fig. S4 shows the effect of varying baseline temperature between 25^o^C and 45^o^C. The results suggest that there are increasing thermal savings as baseline temperatures decline towards those actually observed at australopithecine sites. Under the hairless condition, the only animals to be thermally neutral over the middle of the day are those living in habitats with baseline temperatures of 25^o^C.

Fig. S5 plots the gain from switching to bipedality for the populations in Fig. S4. The relative advantage increases as baseline temperature declines.


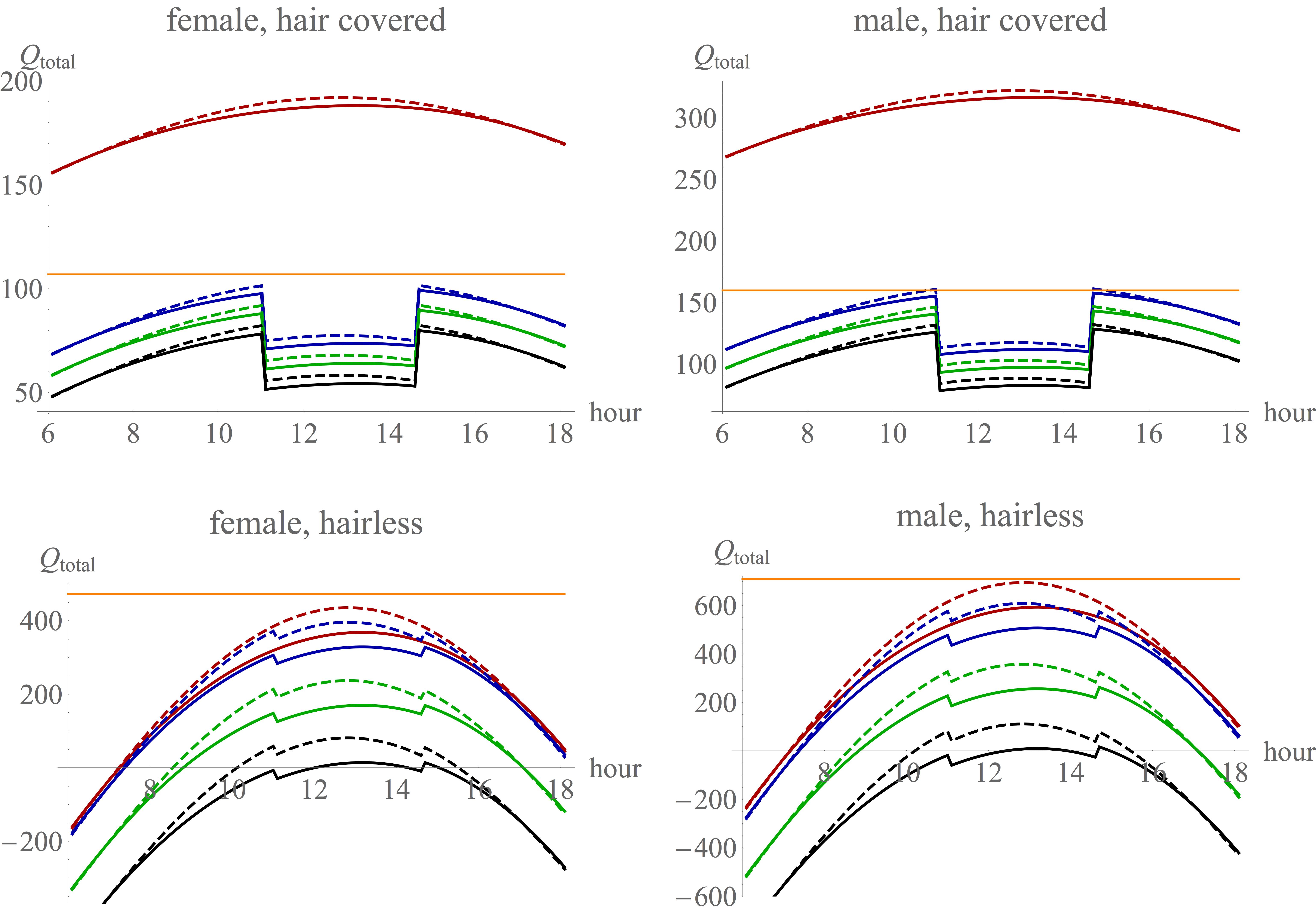


Fig. S4. Sensitivity of the results depicted in Fig. 2 of the paper to the baseline temperature. The combined effect of the re-parameterisation of the Ruxton-Wilkinson (RW) model when walking is limited to 16% of the day and confined to the morning and evening. Red: RW model baseline; blue, green, black: RW model with all three additional factors included, with blue baseline temperature at 45^o^C, green at 35^o^C, and black at 25^o^C. Dash: quadrupedal; continuous: bipedal. Orange: heat dissipation limit.


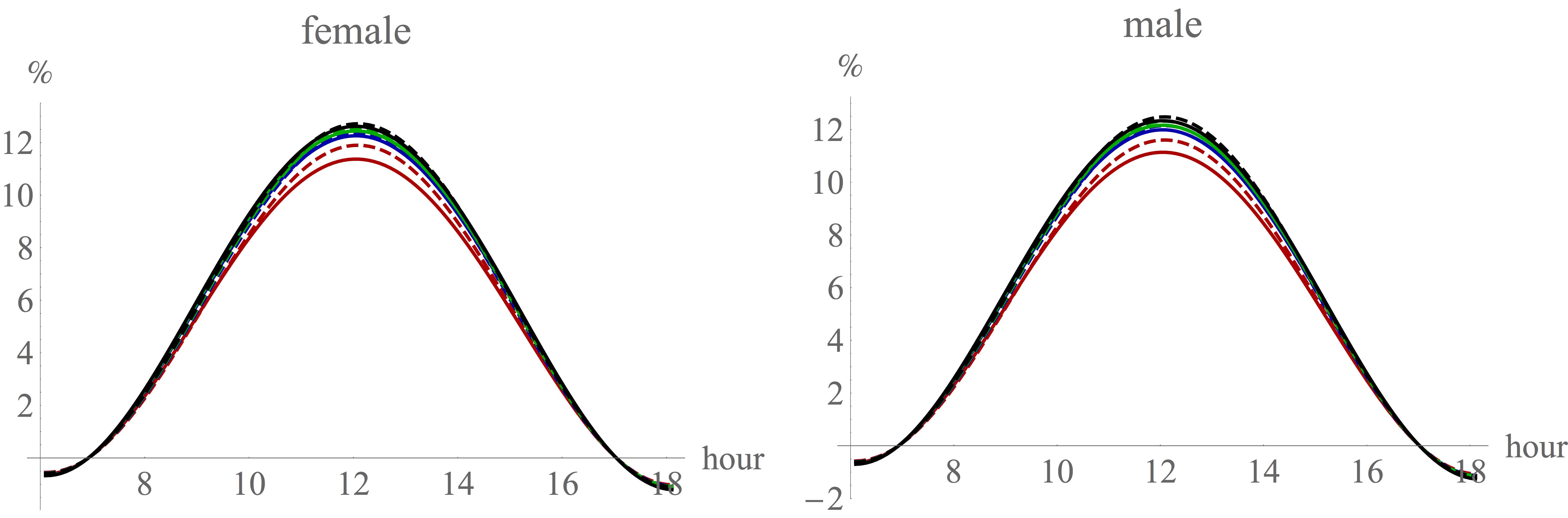


Fig. S5. Sensitivity of the results depicted in Fig. 3 of the paper to the baseline temperature. The percentage advantage from switching from a quadrupedal to a bipedal stance for (a) females and (b) males, respectively. Red: RW model baseline; blue: blue, green, black: RW model with all three additional factors included; with blue baseline temperature at 45^o^C, green at 35^o^C, and black at 25^o^C. Dash: hairless; continuous: hair covered.
